# Candidate genes screening based on phenotypic observation and transcriptome analysis for double flower of *Prunus mume*

**DOI:** 10.1186/s12870-022-03895-0

**Published:** 2022-10-26

**Authors:** Huanhuan Zhu, Yan Shi, Junwei Zhang, Manzhu Bao, Jie Zhang

**Affiliations:** Key Laboratory of Horticultural Plant Biology (Ministry of Education), College of Horticulture and Forestry Sciences, Huazhong Agricultural University, Wuhan, Hubei 430070 China

**Keywords:** *P. mume*, Morphology observation, Double flower, RNA-seq, WGCNA

## Abstract

**Background:**

*Prunus mume* is an early spring flower of Rosaceae, which owns high application value in gardens. Being an excellent ornamental trait, the double flower trait has always been one of the important breeding goals of plant breeders. However, the key regulatory genes of double flower traits of *P. mume* are still unclear at present.

**Results:**

The floral organs’ morphological differences of 20 single and 20 double flower cultivars of *P. mume* were compared firstly. And it was found that double flower trait of *P. mume* were often accompanied by petaloid stamen, multiple carpels and an increase in the total number of floral organs. Then, transcriptome sequencing of two representative cultivars *P. mume* ‘Danban Lve’ and *P. mume* ‘Xiao Lve’ were conducted at 3 Stage of flower bud development with distinct morphological differentiation. 3256 differentially expression genes (DEGs) were detected, and 20 candidate genes for double flower trait of *P. mume* were screened out including hub genes *PmAP1–1* and *PmAG-2* based on DEGs function analysis and WGCNA analysis. And it was found that epigenetic and hormone related genes may also play an important role in the process of double flower.

**Conclusions:**

This study suggested that the double flower trait of *P.mume* is more like accumulation origin based on morphological observation. 20 genes and co-expression network related to the formation of double flower *P. mume* were preliminarily screened through transcriptomics analysis. The results provided a reference for further understanding of the molecular mechanism of double flower trait in *P. mume*.

**Supplementary Information:**

The online version contains supplementary material available at 10.1186/s12870-022-03895-0.

## Background

Double flower refers to the phenomenon that the number of petals or whorls of petals increases [1]. Ornamental plants with double flower are more popular in the flower market which makes double flower more marketable. However, The formation mechanism of double flower is very complicated [2]. Flower development can be subdivided into several steps, starting with floral induction. The key step after the formation of floral meristem is the formation and development of floral organs. Different floral organs are formed in different positions of floral meristem under the regulation of various genes. About 30 years ago, researchers proposed the first model hypothesis to regulate the recognition of different floral organs [3]. The model proposes that the floral organs in the flower meristem from the outside to the inside are determined by three classes of floral organ characteristic genes (called classes A, B and C) or a combination of them, respectively [4]. Among them, C-function gene AGAMOUS (AG) and A-function gene APETALA2 (AP2) were identified as two important genes responsible for double valve formation. And one of the important mechanisms is the functional loss of *AG* [5]. A similar mechanism of double flower formation has been found in *Petunia hybrida*, *Torenia fournieri*, and *P. lannesiana* [6–8]. Besides the loss of *AG* function, Dubois et al. [9] found that the difference of *AG* expression site and expression level may be the reason for the formation of double flower in cultivated roses. In recent years, the A-function gene *APETALA2* (*AP2*) has also been found to be the key gene that causes double flower phenotypes of many ornamental plants, such as *Rosa rugosa*, *P. persica* and *Dianthus chinensis* [10–13]. In addition to the floral organ identity genes, *SEUSS*, *LEUNIG* and *RABBIT EARS* involved in the regulation of *AG* gene had been reported in *Arabidopsis thaliana* to influence the development of floral organs by regulating the expression of floral organ identity genes [14, 15].

Current research shows that the total number of floral organs seem to be closely related to the activity of floral meristem, which was one of the affected factors. In *Nigella damascena*, studies have shown that flowers with larger meristems usually have more organs than flowers with smaller meristems [16]. In Arabidopsis, the negative feedback loop of *WUSCHEL* (*WUS*) and *CLAVATA 3* (*CLV3*) plays a role in maintaining stem cell activity in floral meristem, and inactivation of *WUS* will lead to the reduction of floral meristem and the number of floral organs [17–19]. Some floral meristem size genes, such as *CLV1*, *CLV2* and *WIGGUM*, also affect the number of floral organs by regulating the size of floral meristem [20]. In addition, it was found that *AG* gene controlled the certainty of floral meristem by interacting with *WUS* gene [21, 22]. *FLORAL ORGAN NUMBER 1*, *ULTRAPETALA*, *KNUCKLES* (*KNU*) and *SUPERMAN* had also been reported to affect the number of floral organs by affecting the expression of *WUS* gene [23–26].

At present, the research on the mechanism of double flower *P. mume* mostly focused on the homologous cloning and functional analysis of ABCE genes in model plants [27, 28]. Besides, some candidates for traits related to double lobe had also been preliminary mapped. Zhang [29] excavated two quantitative trait loci controlling the number of petals by using *P. mume* ‘Liu Ban’ × *P. mume* ‘Fentai Chuizhi’ F1 population. Zhang et al. [30] found that a region of about 3.6 Mb on chromosome 1 may be related to the number of petals, carpel traits and bud hole size through genome-wide association study of *P. mume* germplasm resources. Due to the complexity of the regulation mechanism of double flower development, the mechanism of double flower formation of *P. mume* has not yet been solved, and candidates of double flower formation and the regulation network of flower development still need to be further studied.

In this study, the floral organ morphology difference of 40 single and double flower *P. mume* cultivars were compared firstly. Then 2 representative cultivars were selected to seek the differences in floral organ primordia differentiation both on anatomy and transcriptome level. Finally, 20 candidate genes were screened out and the gene network of 3 hub genes were conducted. This study provide insight into the anatomy and organ differentiation of single and double flower trait in *p.mume* and is supposed to lay a foundation for the subsequent disclosure of the molecular mechanism for double flower trait of *P. mume*.

## Results

### Flower morphology difference between single and double cultivars of *P. mume*

**Fig. 1:**
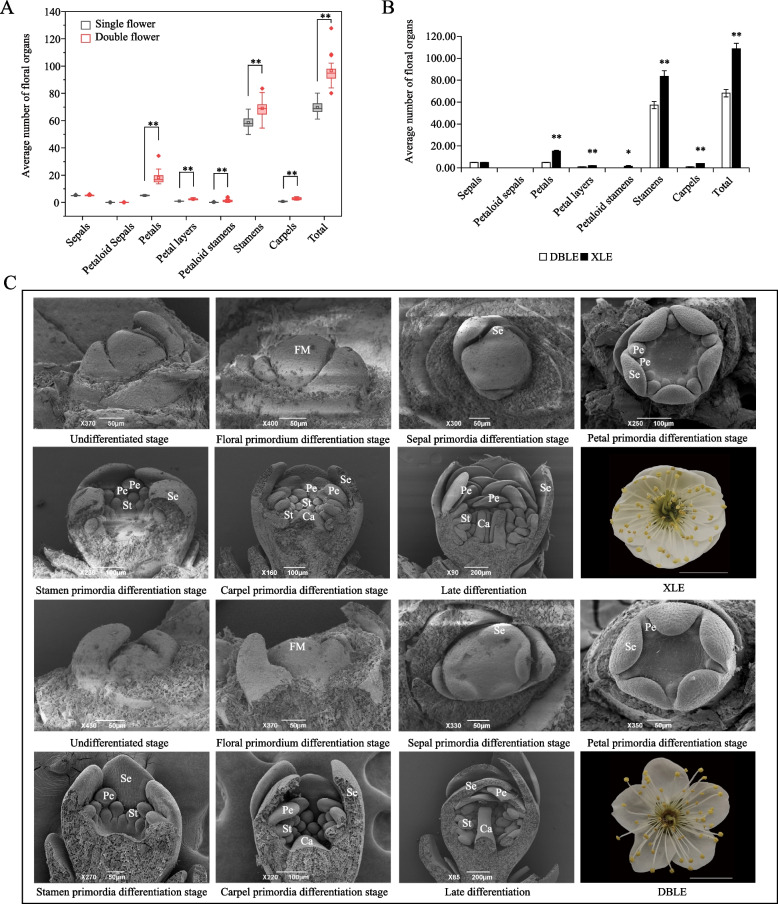
Floral organ morphological differences between *P. mume* ‘Danban Lve’ (DBLE) and *P. mume* ‘Xiao Lve’ (XLE). **A** Comparison of the number of floral organs between single and double flower cultivars. **B** Comparative analysis of the number of floral organs of DBLE and XLE. **C** and **D** are scanning electron microscope images of flower bud primordia differentiation of XLE and DBLE, respectively. FM, Se, Pe, St and Ca represent flower primordium, sepals, petals, stamens and carpels. Asterisks indicate statistically significant differences (Student’s *t* test *P* value, *P < 0.05, **P < 0.01)

Firstly, the number of floral organs of 20 single and 20 double flower cultivars were counted. Based on Fig. 1A, no significant difference were obseved in the average number of sepals and petaloid sepals between single and double flower cultivars However, the phenomenon of petaloid sepals and petaloid stamens existed in both single and double flower cultivars. The average number of petaloid stamens and stamens in double flower cultivars was significantly higher than that in single flower cultivars. A highly significant difference (*P* < 0.01) in the number of petals was also detected between single and double flower, with the average number of petals for single flower cultivars was 5.14 ± 0.20, while it was 18.28 ± 4.82 for double flower cultivars. And the average number of carpels in double flower was also significantly higher than that in single flower (Fig. 1A). As for the total number of floral organs, it was significantly higher in double flower cultivars than that in single flower cultivars, with the average number of floral organs in double flower cultivars being 96.66 ± 9.90, while it was 69.69 ± 5.15 in single flower cultivars (Fig.1A).

Among the 40 cultivars, DBLE and XLE in relationship were near with each other [31, 32]. Except for the number of petals, the other characters were basically the same in the two cultivars. Further analysis was carried out on these two cultivars. The number of floral organs between single flower cultivar *P. mume* ‘Danban Lve’ (DBLE) and double flower cultivar *P. mume* ‘Xiao Lve’ (XLE) was consistent with that of other cultivars (Fig. 1B). Then, the morphological differentiation processes of flower buds of DBLE and XLE were observed by scanning electron microscope (SEM). It was found that the morphological differentiation of the two cultivars began at the petal primordia differentiation stage (Fig. 1C). XLE formed one more whorl of petal primordia than DBLE. After the petal primordia was formed, it stretched gradually, and the petal sheet structure could be clearly seen at the carpel primordia differentiation stage (Fig. 1C).

### Transcriptome analysis of flower bud in DBLE and XLE

In order to explore the gene expression differences and changes during the formation of single and double flowers of *P. mume*, the transcriptome sequence of flower buds in three different stages of petal differentiation between XLE and DBLE were conducted, namely, the petal primordia differentiation stage (S1), stamen primordia differentiation stage (S2) and carpel primordia differentiation stage (S3). The results of raw data quality control and filtering indicated that the proportion of clean reads in all samples was more than 95%, and the percentage of Q30 bases was more than 93.00%. After comparison with the reference genome [33], a total of 32,844 genes were obtained. Five databases were used to annotate gene functions, and the NR annotation ratio was the highest, reaching 93.48% (Table S1).

A total of 7810 comparison combinations DEGs were obtained. The process of flower bud morphological observation suggested that the morphology of petal primordia changed gradually with time (Fig. 1C), so the maSigpro method [34, 35] was further used to analyze the time-series DEGs of XLE and DBLE in S1-S3, and a total of 4868 time-series DEGs were further selected. Then, we focused on 3256 DEGs that which were differentially expressed among the comparison combinations and in time-series. 12 DEGs were randomly selected for qRT-PCR analysis and the correlation analysis showed that the correlation coefficient of 11 genes was greater than 0.76 (*P* < 0.1) (Fig. S1), and linear regression analysis showed that the overall correlation coefficient between qRT-PCR and the expression trend of transcriptome data was 0.75 (*P* < 0.01).

**Fig. 2:**
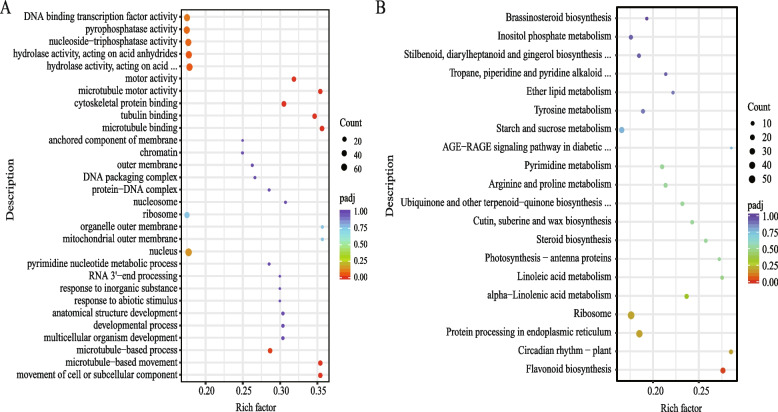
Differential gene enrichment analysis of single and double flower *P. mume*. **A** and **B** are bubble charts of GO and KEGG enrichment analyses of DEGs between DBLE and XLE, respectively. A dot represents a GO Term /KEGG [36–38] pathway, the size of the dot represents the number of genes annotated to the GO Term /KEGG pathway, and the color from red to purple represents the significance of enrichment. Rich factor means the ratio of the number of differential genes annotated to the pathway term to the total number of genes annotated to the Pathway term

To explore the function of DEGs, 3256 DEGs were subjected to Gene ontology (GO) enrichment analysis. The results showed that DEGs were significantly enriched in microtubule-based movement (GO: 0007018) and movement of cell or subcellular component (GO: 0006928) of Biological Process category. And in Molecular Function category, it was also significantly enriched in five terms related to microtubule and movement, which were microtubule binding (GO: 0008017), tubulin binding (GO: 0015631), microtubule motor activity (GO: 0003777), cytoskeletal protein binding (GO: 0003777) and motor activity (GO: 0003774), respectively, and the rich factor was relatively high (Fig. 2A). In addition, multicellular organism development (GO: 0007275) and response to abiotic stimulation (GO: 0009628) also have a relatively high rich factor. KEGG enrichment analysis showed that 3256 DEGs were enriched to 118 metabolic pathways, which were significantly enriched in flavonoid biosynthesis (pmum00941) (Fig. 2B). And the DEGs were also enriched in the circadian rhythm-plant (pmum04712), alpha-linolenic acid metabolism (pmum00592) and photosynthesis-antenna proteins (pmum00196) pathways.

### DEGs analysis related to the formation of double flower

**Fig. 3:**
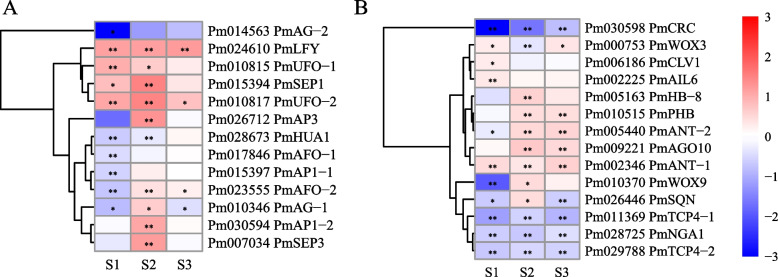
Candidate DEGs expression patterns related to floral organ recognition (**A**) and floral meristems maintenance and termination (**B**). The data used for heat mapping is log_2_Foldchange, in which red indicates that the expression level of this gene in XLE is higher than that in DBLE, while blue is opposite. Asterisks indicate statistically significant differences (Negative binomial distribution *P*-adjust value, *P < 0.05, **P < 0.01)

Since floral organ recognition gene and the activity of floral meristem determines the identity of the floral organs and the total number of floral organs, these two types of genes are important in flower development [4, 26] 13 DEGs related to floral organ recognition and development were identified through homologous comparison with model plants, including 7 floral organ identity genes. The expression patterns of 2 A-function genes *PmAP1–1* (*Pm015397*) and *PmAP1–2* (*Pm030594*) were slightly different (Fig. 3A). *PmAP1–1* was significantly down-regulated in S1 stage of double flower XLE compared with single flower DBLE, while *PmAP1–2* was significantly up-regulated in S2 stage of XLE. The expression patterns of 2 C-function genes *PmAG-1* (*Pm010346*) and *PmAG-2* (*Pm014563*) were also different, but both were significantly downregulated in S1 stage of XLE. Interestingly, in addition to the *AG* gene, *Pm028673* was identified as the gene *ENHANCER OF AG-4 protein 1* (*HUA1*) which can enhance the mutant phenotype of *AG* [39], and it was significantly downregulated in S1 and S2 stages of XLE. In addition, 5 DEGs which may regulate the gene expression of floral organ identity genes were identified, namely, 2 *ABNORMAL FLORAL ORGANS* (*AFO*, *Pm017846* and *Pm023555*), 2 *UNUSUAL FLORAL ORGANS* (*UFO*, *Pm010815* and *Pm010817*) and *LEAFY* (*LFY*, *Pm024610*) genes.

14 DEGs associated with floral meristem maintenance and termination were identified. The expression patterns of two *WUS* family genes *WUSCHEL RELATED HOMEOBOX 3* (*WOX3*, *Pm000753*) and *WOX9* (*Pm010370*) were almost opposite (Fig. 3B). *PmWOX3* was significantly upregulated in S1 and S3 of XLE compared with DBLE and significantly downregulated in S2 stage (Fig. 3B). *Pm030598* was identified as *CRABS CLAW* (*CRC*) and its expression is significantly downregulated during S1-S3 stages of XLE. Meanwhile, 5 DEGs were identified as *ARGONAUTE10* (*AGO10*, *Pm009221*), *PHABULOSA* (*PHB*, *Pm010515*), *HOMEOBOX GENE 8* (*HB-8*, *Pm005163*) and *AINTEGUMENTA* (*ANT*, *Pm002346* and *Pm005440*), and significantly upregulated in S2 and S3 stages of XLE.

**Fig. 4:**
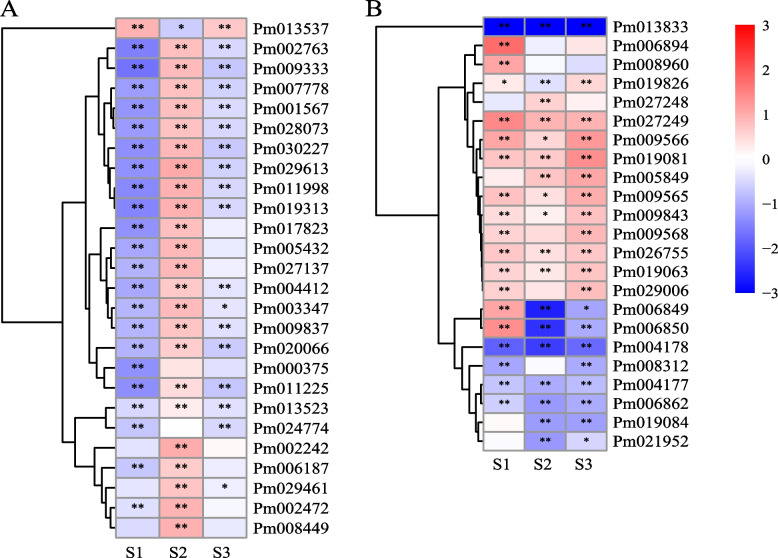
Candidate DEGs expression patterns related to microtubule movement (**A**) and flavonoid biosynthesis (**B**). The data used for heat mapping is log_2_FoldChange, in which red indicates that the expression level of this gene in XLE is higher than that in DBLE, while blue is opposite. Asterisks indicate statistically significant differences (Negative binomial distribution *P*-adjust value, *P < 0.05, **P < 0.01)

Besides, since microtubule and flavonoid biosynthesis related terms were enriched based on GO and KEGG analyses of DEGs, genes related to these two biological processes were further analyzed. 26 genes related to microtubule were detected in DEGs, of which 22 were kinesin family genes. Active differential expressions of microtubule-related genes were observed in S1-S3 stages, especially in S1 and S2, with 23 and 24 genes differentially expressed, respectively (Fig. 4A). Except for *Pm013537*, the expression patterns of other genes were almost identical, which were significantly downregulated in S1 and S3 stages of XLE, but significantly upregulated in S2 stage. A total of 23 genes were identified in the flavonoid biosynthesis pathway, and there were also active expression differences in S1-S3 stages (Fig. 4B). Among them, 13 genes were upregulated in XLE, including *Cytochrome P450 CYP73A100* gene (*Pm008960*) and 3 *Polyketide synthase 5* genes (*Pm009566*, *Pm09565* and *Pm09568*) located upstream of the pathway.

**Fig. 5:**
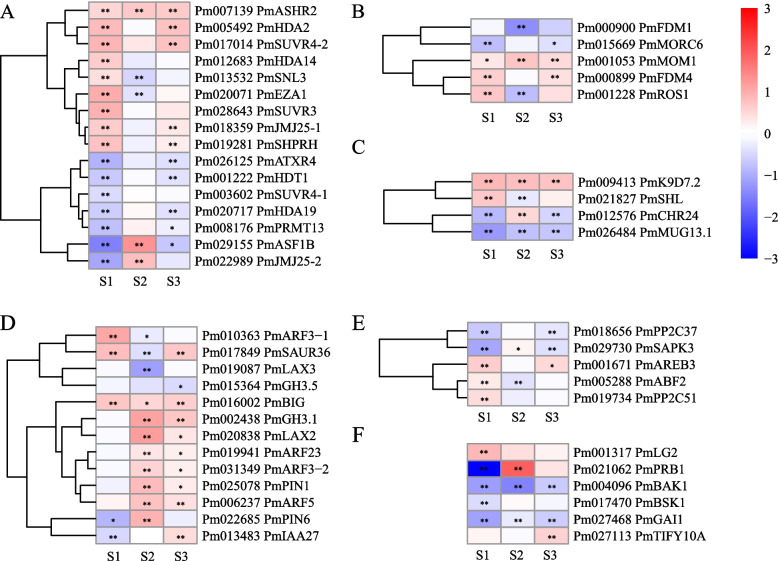
The expression patterns of candidate DEGs associated with epigenetics and hormone. The figure shows the expression patterns of genes related to histone modification (**A**), DNA methylation (**B**), chromatin remodeling (**C**), auxin (**D**), abscisic acid (**E**) and other hormone-related genes (**F**) respectively. The data used for heat mapping is log_2_FoldChange, in which red indicates that the expression level of this gene in XLE is higher than that in DBLE, while blue is opposite. Asterisks indicate statistically significant differences (Negative binomial distribution *P*-adjust value, *P < 0.05, **P < 0.01). S1: the petal primordia differentiation stage, S2: stamen primordia differentiation stage, S3: carpel primordia differentiation stage (S3)

Further analysis of the annotation results of DEGs functions revealed 25 epigenetic regulation and 24 hormone-related DEGs. Among the epigenetic-related genes, 16 histone modification-related genes were significant differentially expressed between DBLE and XLE at S1 stage (Fig. 5A). 9 genes were upregulated and 7 genes were downregulated in XLE compared with DBLE. At the same time, a few DEGs related to methylation and chromatin remodeling were detected, and there were active differential expressions in all three stages (Fig. 5B and C). As for the plant hormone-related genes, auxin-related genes were the most, including 8 auxin response-related genes and 5 auxin polarity-related genes (Fig. 5D). And auxin-related genes were mostly differentially expressed in S2 and S3 stages of DBLE and XLE, among which 7 genes were significantly upregulated in XLE compared with DBLE, including *Auxin response factor 5* (*ARF5*, *Pm006237*), *PmARF3–2* (*Pm031349*), *Auxin efflux carrier component 1* (*PIN1*, *Pm025078*) and *Auxin transporter-like protein 2* (*LAX2*, *Pm020838*) (Fig. 5D). 5 DEGs were involved in abscisic acid signal transduction pathway (Fig. 5E). In addition, a few DEGs related to salicylic acid, brassinolide, gibberellin and jasmonic acid were identified (Fig. 5F).

### Gene co-expression network analysis

**Fig. 6:**
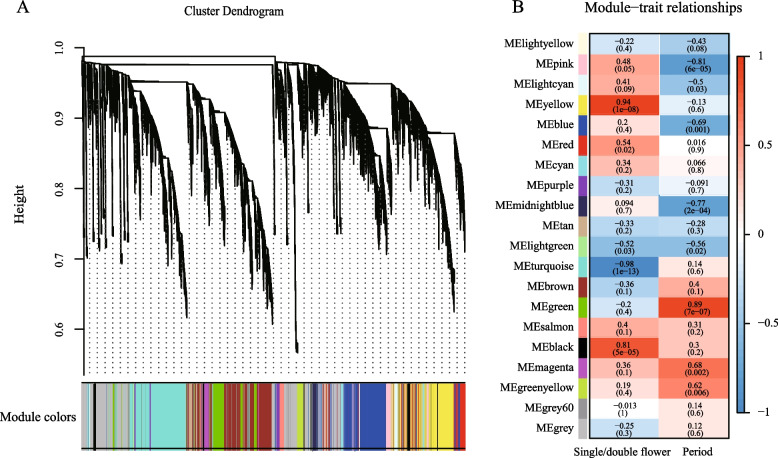
Dynamic division of WGCNA module and correlation analysis with traits. **A** The dynamic division of WGCNA modules, with different colors representing different modules, Y-axis (Height) is the cluster distance between two nodes (genes), and the distance in X-axis is meaningless; **B** The heatmap of correlation analysis between different modules and traits. The numbers in brackets are the results of significance analysis (Student’s *t* test *P* value)

The flower development process is usually controlled by interacting gene networks [40]. To further screen the key hub genes during the double flower formation of *P. mume* and analyze their co-expression gene networks, the weighted gene co-expression network analysis (WGCNA) method was conducted. 20 modules were obtained after dynamic cutting (Fig. 6A). The correlation analysis between single and double flower phenotypes and modules showed that yellow, turquoise and black modules were significantly correlated with double flower (*R* > 0.65, *P* < 0.05) (Fig. 6B). While the correlation analysis between developmental stages and modules indicated that green, pink, midnightblue, blue and magenta modules were significantly correlated with the developmental stage (*R* > 0.65, *P* < 0.05).

Further, the hub genes were determined by KME value, and 2 floral organ identity genes (*PmAG-1* and *PmAP1–2*) and 1 floral meristem-related gene (*AINTEGUMENTA-LIKE 6*, *Pm002225*) were screened out. In addition, 3 epigenetic-related genes *PmMORC6 (MICRORCHIDIA 6*, *Pm015669)*, *PmROS1* (*Repressor of silencing 1*, *Pm001228*) and *PmSHL* (*SHORT LIFE*, *Pm021827*) and 4 auxin-related genes (*PmARF5*, *PmARF3–2*, *PmPIN1* and *PmLAX2*) were identified.

**Fig. 7:**
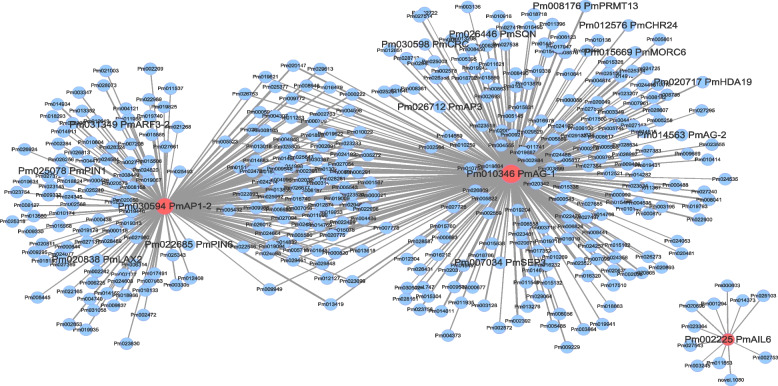
Co-expression DEGs network of *PmAG-1*, *PmAP1–2* and *PmAIL6* revealed by WGCNA method. Each dot represents a gene, in which the size of the dot represents the connectivity of genes, and the larger the dot, the greater the connectivity. The red dots indicate key hub genes. Each edge in the figure represents the regulatory relationship between genes

Since *PmAG-1*, *PmAP1–2* and *PmAIL6* may play an important role in organ recognition and meristem activity maintenance, in order to further explore their functions in the development of double flower, co-expression gene networks of these 3 genes were further analyzed. It was found that PmAG-1 and *PmAP1–2* formed the main network together, and 388 DEGs were co-expressed with them, of which 96 genes were co-expressed with *PmAG-1* and *PmAP1–2* at the same time. And *PmAIL6* was co-expressed with 11 genes alone (Fig. 7). GO enrichment analysis of network gene suggested that 96 genes co-expressed with both *PmAG-1* and *PmAP1–2* and those genes only co-expressed with *PmAP1–2* were significantly enriched in microtubule-related and hydrolase activity-related items (Table S2). Genes only co-expressed with *PmAG-1* were significantly enriched in multicellular biological development and fatty acid biosynthesis process. No significantly enriched items were detected as for *PmAIL6* (Table S2).

Further analysis of DEGs in the network showed that genes related to floral meristem, *PmCRC* and *PmSQN* (*SQUINT*, *Pm026446*), were co-expressed with *PmAG-1.* And three floral organ identity genes, *PmAG-2*, *PmAP3* and *PmSEP3*, were co-expressed only with *PmAG-1* (Fig. 7). Interestingly, 4 epigenetic-related genes *PmMORC6*, *PmPRMT13* (*Probable histone-arginine methyltransferase 1.3*, *Pm008176*), *PmHDA19* (*Histone deacetylase 19*, *Pm020717*) and *PmCHR24* (*CHROMATIN REMODELING 24*, *Pm012576*) were also directly co-expressed with *PmAG-1*. 4 auxin-related genes (*PmPIN1*, *PmPIN6*, *PmLAX2* and *PmARF3–2*) were directly co-expressed with another hub gene *PmAP1–2*. 294 variant loci were detected in the 20 candidate genes mentioned above, including 261 SNPs and 22 InDel. Among them, 28 non-synonymous variants were located on *PmAP3*, *PmAIL6*, *PmSQN*, *PmARF5*, *PmARF3–2*, *PmMORC6*, *PmROS1*, *PmCHR24*, which maybe the reason for amino acid changes resulting in floral meristematic tissue changes, auxin and epigenetic response.

## Discussion

There are various origins for the formation of double flowers. The original species of *P. mume* is usually a single flower with 5 petals, and during the long-term cultivation, double flower cultivars gradually appear [41]. Based on the statistical analysis of the floral organs numbers in 40 *P. mume* cultivars, it was found that the number of petaloid stamens in double flower cultivars was significantly higher than single flower (Fig. 1A). Compared with *P. lannesiana* and rose, whose double flower traits are caused by the transformation of stamens into petals and the number of stamens is correspondingly reduced [7, 9, 42], the number of stamens of double flower was significantly higher than that of single flowers in *P.mume* (Fig. 1A). Interestingly, not only the number of petals and stamens, but also the number of carpels and the total number of floral organs in double flower cultivars were significantly higher than those in single flower cultivars. A similar double flower phenotype has been reported in *D. chinensis*, which was reported to be related to the abnormal activity of floral meristem [12]. The total number of floral organs was also related to the activity of floral meristem in *Arabidopsis* [16, 43, 44]. To sum up, the double flower *P. mume* is more like accumulation origin, and it may be related to the difference of floral meristem activity.

Transcriptome provides a global analysis of gene expression and molecular basis for analyzing biological processes. In this study, transcriptome analyses of flower buds in the morphological differentiation stages of *P. mume* were conducted for the first time. DEGs between single and double flower were significantly enriched in microtubule-related items and flavonoid biosynthesis pathway Interestingly, it was shown that microtubule tissue can regulate the anisotropic shape of petals during the formation of petals [45], while flavonoids regulate auxin transportation and metabolism, and can also integrate auxin with other hormones, reactive oxygen species, transcription regulation and other signaling pathways [46]. The persistent significantly different expression of genes related to microtubule and flavonoid biosynthesis (Fig. 4) may play an important role in the formation of double flower in *P. mume*.

Functional and WGCNA analysis of DEGs screened 20 possible candidates which need further attention. 2 floral organ identity genes (*PmAG-1* and *PmAP1–2*) and 1 floral meristem-related gene (*AINTEGUMENTA-LIKE 6*, *Pm002225*) were important. Since genes related to floral organ recognition determine the floral organ types of primordial differentiation, which is essential for the development of floral organs [4]. And the activity of floral meristem is closely related to the total number of floral organs [43]. Ma et al. [47] found that the increase of the methylation level of *RhAG* promoter may lead to the low expression of *RhAG*, and then lead to the increase of rose petals. Previous studies also shown that histone modification plays an important role in the determination and development of floral organs and the termination of floral meristem [48, 49]. Differential expression the 3 epigenetic-related genes *PmMORC6*, *PmROS1* and *PmSHL* may influence the floral meristem development of double flowers in *P. mume* (Fig. 5A). Besides, the identified auxin-related genes (*PmARF5*, *PmARF3–2*, *PmPIN1* and *PmLAX2*) may related to the development of double flower since studies have shown that the direction of transport caused by auxin polar transport can provide the required auxin concentration gradient for cell expansion [50]. Meanwhile, the *ARF* gene was a key candidate hub gene for the development of double flower in *Malus spectabilis* [51].

The formation, differentiation and development of double flower have complex regulatory mechanisms, and the flower development process in plants is usually controlled by interacting gene networks [2, 40]. The network analysis of hub genes *PmAG-1* and *PmAP1–2* found that, the genes co-expressed with *PmAG-1* and *PmAP1–2* were significantly enriched in microtubule-related items coincidentally (Table S2). The floral organ identity genes *PmAG-2*, *PmAP3* and *PmSEP3* were co-expressed with *PmAG-1,* and the genes *PmCRC* and *PmSQN*, which are upstream and downstream of *AtAG* and regulate flower meristem termination in *A. thaliana*, were also co-expressed with *PmAG-1*, suggesting that they may co-regulate double flower formation (Fig. 7).

## Conclusions

Overall, this study observed the morphological differences between single and double flower of 40 representative cultivars in *P. mume* and the origin of double flowers of *P. mume* was preliminarily discussed. Then, the transcriptome of early flower buds of DBLE and XLE were analyzed, with microtubule-related activities and flavonoid biosynthesis were enriched. 20 candidate genes, including 10 hub genes and 10 genes co-expressed with *PmAG-1* and *PmAP1–2,* need further attention. In all, the analyses of the comprehensive transcriptome data set in this study provide a useful genomic resource for *P.mume*, and molecular insights into the the mechanism of double flower traits at transcriptomic level.

## Methods

### Plant material

The 20 single flower and 20 double flower *P. mume* cultivars (Fig. S2, Table S3) used for counting the number of floral organs were collected in Chinese *P.mume* Germplasm Bank in Moshan Mei Flower Garden, Hubei Province (30°33′ N; 114°24′ E) in February 2021. Each cultivar randomly collected 10 flowers in different directions and branches in full bloom. Transcriptome sequencing used flower buds of DBLE and XLE, which were collected from July to November 2020 in Moshan Mei Flower Garden. Flower buds of the same shape and size were collected every 7 d in duplicate, one was used for anatomical observation of the development period, and the other was instantly frozen in liquid nitrogen and stored at − 80 °C. Each cultivar collected flower buds in 3 stages: petal primordia differentiation (S1), stamen primordia differentiation (S2) and carpel primordia differentiation (S3). Every 20 flower buds were mixed as one repeat, and each cultivar collected three biological repeats in each stage.

### Scanning electron microscope

Flower buds of DBLE and XLE were collected every 7 d in Moshan Mei Flower Garden from July to November 2020. After observing the development period of flower buds under the stereomicroscope, the dissected flower buds were immediately stored in 2.5% glutaraldehyde phosphate buffer (pH 7.0, used for SEM). After the sample was fixed for 24 h, it was dehydrated by alcohol and isoamyl acetate, dried at the critical point of CO2, adhered to a platform and coated with gold, and then analyzed by JSM-6390 LV (Hitachi, Japan) electron microscope (microscope platform of Huazhong Agricultural University) [52].

### Transcriptome sequencing

The flower bud samples of DBLE and XLE in three periods were entrusted to Novogene Company (Tianjin, China) for sequencing on Illumina HiSeq 4000 platform. Raw data of fastq format were firstly processed through FASTP (version 0.19.7) software, and the parameters are: fastp -g -q 5 -u 50 -n 15 -l 150. Then, clean data were obtained by removing reads containing adapter, reads containing N base (N indicates that the base information could not be determined) and low-quality reads (reads with Qphred≤20 bases accounting for more than 50% of the entire read length) from raw data. All the downstream analyses were based on clean data with high quality. Index of the reference genome was built using Hisat2 v2.0.5 and paired-end clean reads were aligned to the reference genome using Hisat2 v2.0.5. The new gene was predicted by StringTie v1.3.3b [53].

### Difference analysis and enrichment analysis

FeatureCounts v1.5.0-p3 was used to count the reads numbers mapped to each gene. And then the fragments per kilo bases per million reads (FPKM) of each gene were calculated based on the length of the gene and read count mapped to this gene. Differential expression analysis of two conditions/groups (two biological replicates per condition) was performed using the DESeq2 R package (1.20.0). The gene expression of each combination between the same period of XLE and DBLE, the adjacent period of DBLE and the adjacent period of XLE were compared and analyzed respectively. The resulting *P*-values were adjusted using Benjamini and Hochberg’s approach for controlling the false discovery rate. Comparison combinations DEGs were obtained according to fold change (FC) > 1.5 and *P*-adj < 0.05. Time-series difference analysis was carried out based on the Next maSigPro package (version 1.6.0) of R 4.0.4 [30]. ClusterProfiler (3.4.4) R package was used to realize GO and KEGG enrichment analysis of DEGs.

### qRT-PCR analysis

In this study, the qRT-PCR was carried out using samples consistent with transcriptome sequencing. The *Pm006362* gene of *P. mume* was used as the internal reference gene [54]. Using Primer Premier 5 software, the specific primers of the target gene for qRT-PCR were designed and synthesized by Qingke company (Wuhan, China). See Table S4 for primer sequences of the internal reference gene and target gene. qRT-PCR amplification was performed by ABI 7500 real-time quantitative PCR instrument (Applied Biosystems, CA, USA). The quantitative real-time PCR assay mix (20 μL) consisted of 3 μL cDNA sample 1 μL TB Green Premix Ex Taq II (TaKaRa，Japan)，1 μL ROX Reference Dye (TaKaRa，Japan), 3.5 μL of each primer and 8 μL distilled deionized H_2_O. The amplification conditions were 95 °C for 30s, followed by 40 cycles of denaturation at 95 °C for 5 s, annealing at 60 °C for 34 s, and elongation at 95 °C for 15 s，60 °C for 60s，95°C for 15 s. The 2^-ΔΔCT^ values were used to quantify the expression levels of the tested genes [55].

### WGCNA analysis

The weighted co-expression gene network [56] was constructed by using the WGCNA v1.13 package based on R language 4.0.4. According to pickSoftThreshold function, the soft threshold was confirmed, and the best soft threshold in this study was 9. Then, the topological overlap dissimilarity measure was used to calculate the degree of association between genes. The characters were transformed into numerical data, and the Pearson correlation coefficient was used to analyze the correlation between traits and modules. The hub gene is the gene with the most connection points in each module, and its height is expressed by kME (Module Eight Gene-based Connectivity). According to the value of |kME|, the first 30 genes of each module were selected as hub genes. By using the software Cytoscape 3.7.2 for visual display, each node in the network represented a gene, and each edge represented the regulatory relationship between genes [57].

## Supplementary Information

**Additional file 1.**

**Additional file 2.**

**Additional file 3.**

**Additional file 4.**

**Additional file 5.**

**Additional file 6.**

## Data Availability

All data generated or analyzed during this study are included in this published article and its supplementary information files. The RNA-seq data have been deposited in the NCBI Sequence Read Archive, accession number: PRJNA847636.

## Abbreviations

DEGs: Differentially expressed genes
FC: Fold change
FPKM: Fragments per kilo bases per million reads
GO: Gene Ontology
KEGG: Kyoto Encyclopedia of Genes and Genomes
qRT-PCR: Quantitative Real-time PCR
SEM: Scanning electron microscope
WGCNA: Weighted correlation network analysis
