# Supplementary figures and images for: Candidate genes screening based on phenotypic observation and transcriptome analysis for double flower of Prunus mume
Source: BMC Plant Biol. 2022 Oct 26;22:499. doi: 10.1186/s12870-022-03895-0 (PMC9597982; doi:10.1186/s12870-022-03895-0)

Relative expression levels by qRT-PCR

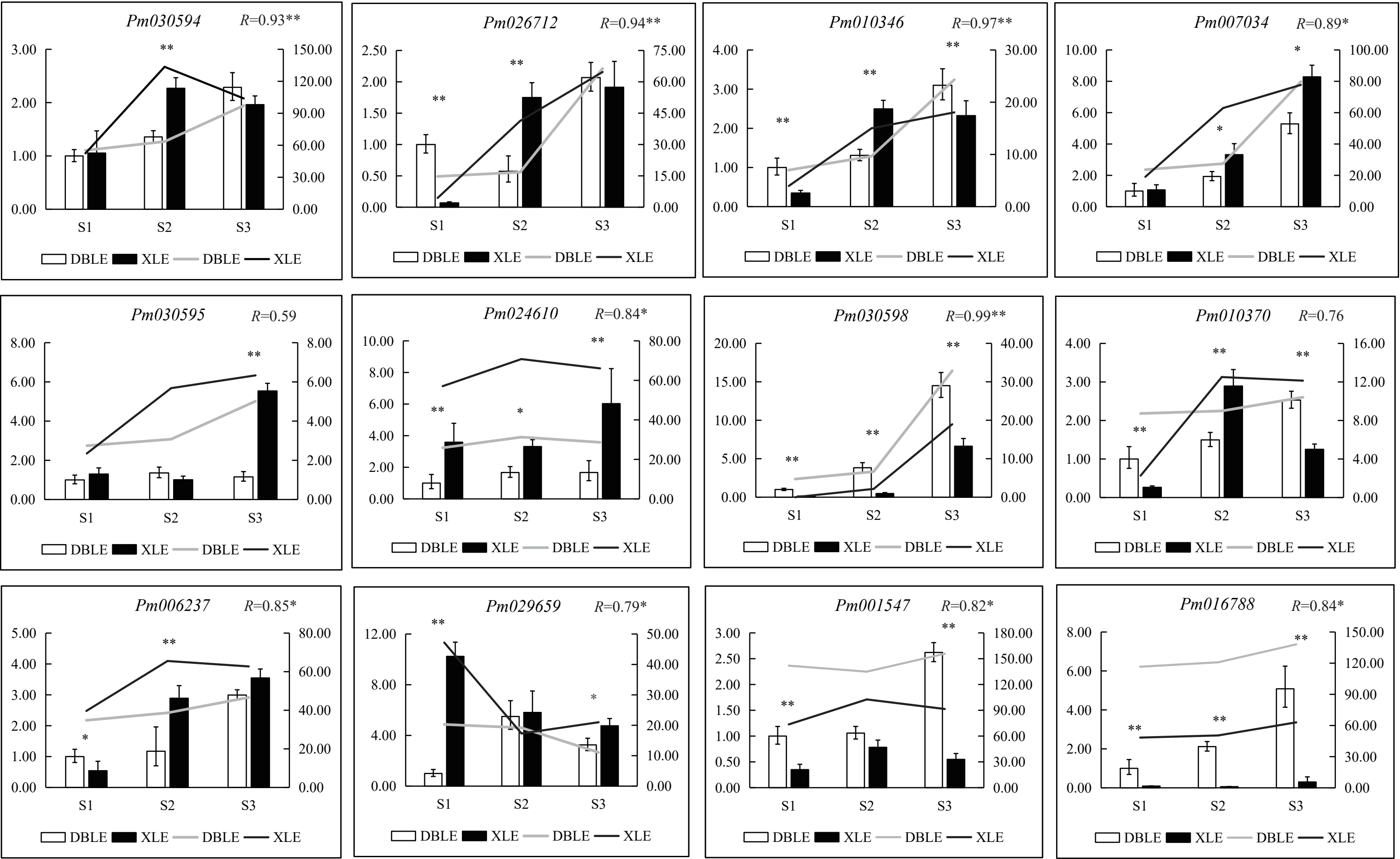

FPKM of RNA-seq

Supplement: Supplementary file 1 — Additional file 1. [file 12870_2022_3895_MOESM1_ESM.pdf]
