# Supplementary material for: Candidate genes screening based on phenotypic observation and transcriptome analysis for double flower of Prunus mume
Source: BMC Plant Biol. 2022 Oct 26;22:499. doi: 10.1186/s12870-022-03895-0 (PMC9597982; doi:10.1186/s12870-022-03895-0)
Supplement: Supplementary file 2 — Additional file 2. [file 12870_2022_3895_MOESM2_ESM.pdf]

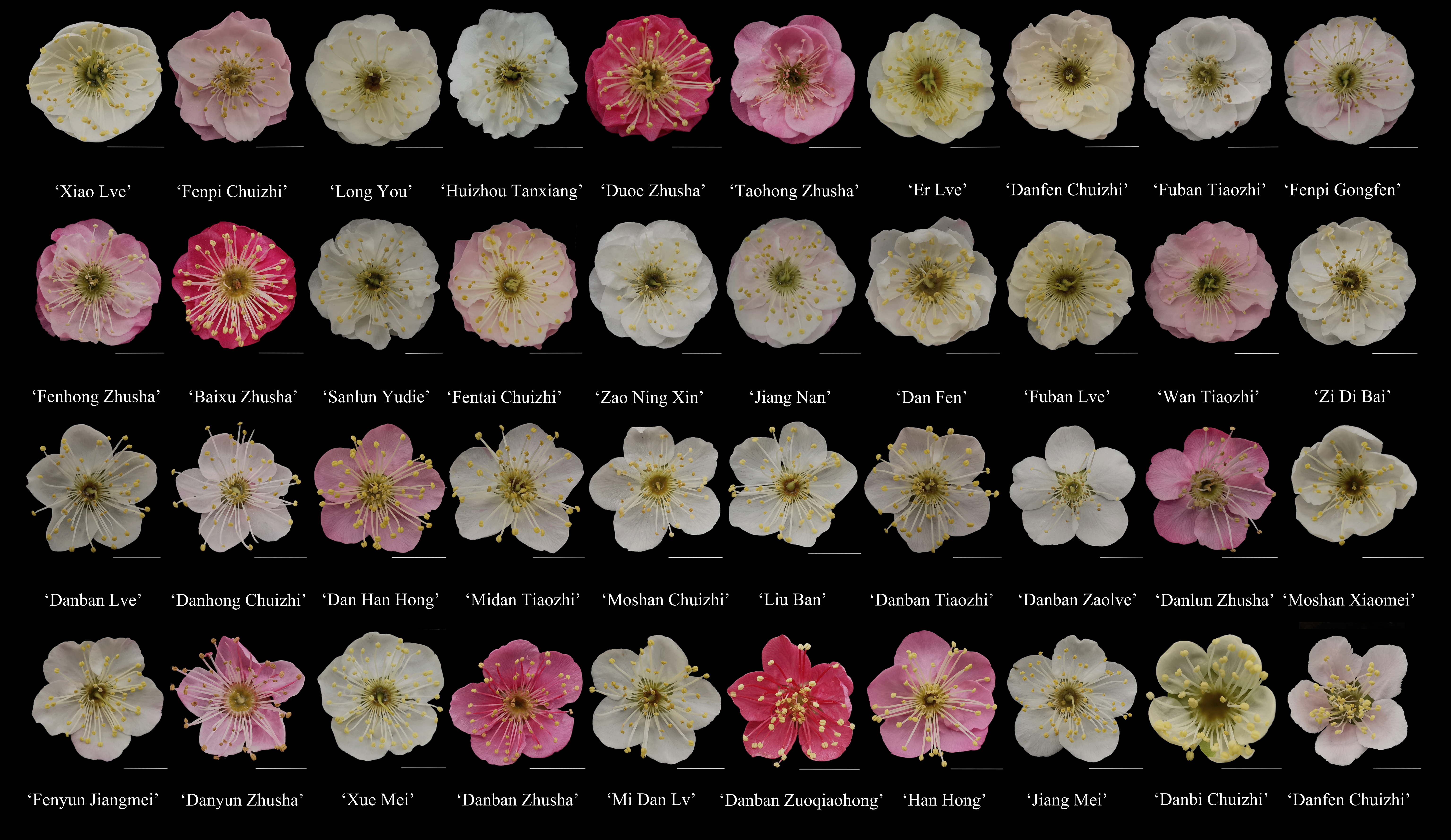
‘Xiao Lve’
‘Fenpi Chuizhi’
‘Long You’
‘Huizhou Tanxiang’
‘Duoe Zhusha’
‘Taohong Zhusha’
‘Er Lve’
‘Danfen Chuizhi’
‘Fuban Tiaozhi’
‘Fenpi Gongfen’
‘Fenhong Zhusha’
‘Baixu Zhusha’
‘Sanlun Yudie’
‘Fentai Chuizhi’
‘Zao Ning Xin’
‘Jiang Nan’
‘Dan Fen’
‘Fuban Lve’
‘Wan Tiaozhi’
‘Zi Di Bai’
‘Danban Lve’
‘Danhong Chuizhi’
‘Dan Han Hong’
‘Midan Tiaozhi’
‘Moshan Chuizhi’
‘Liu Ban’
‘Danban Tiaozhi’
‘Danban Zaolve’
‘Danlun Zhusha’
‘Moshan Xiaomei’
‘Fenyun Jiangmei’
‘Danyun Zhusha’
‘Xue Mei’
‘Danban Zhusha’
‘Mi Dan Lv’
‘Danban Zuoqiaohong’
‘Han Hong’
‘Jiang Mei’
‘Danbi Chuizhi’
‘Danfen Chuizhi’
